# Lethal *Mycobacterium massiliense* Sepsis, Italy 

**DOI:** 10.3201/eid1406.080194

**Published:** 2008-06

**Authors:** Enrico Tortoli, Rita Gabini, Irene Galanti, Alessandro Mariottini

**Affiliations:** * Careggi Hospital, Florence, Italy; † San Donato Hospital, Arezzo, Italy

**Keywords:** Mycobacterium, Mycobacterium massiliense, bacteremia, case report, immunocompromised, letter

**To the Editor:** A strain of *Mycobacterium massiliense* was isolated from the blood of a kidney transplant patient in Italy at the same time she was diagnosed as having pulmonary tuberculosis. *M. massiliense* bacteremia appears to have played a role in her sudden death.

The patient, a 63-year-old woman who had had a kidney transplant 10 years earlier and was receiving immunosuppressive treatment with cyclosporine, azathioprine, and prednisolone, was hospitalized in an intensive care unit because of septic shock, a stuporous condition, hypotension, respiratory insufficiency, and acute renal failure. Results of initial microbiologic investigations (cultures of blood, urine, and bronchial aspirate, and nasal and pharyngeal swabs; tests for antigens of *Legionella pneumophila*, pneumococcus, and *Cryptococcus neoformans*; and serologic analysis for pneumotropic viruses and bacteria) were negative.

Hematologic analysis showed leukopenia with high neutrophil counts. Septic shock–specific therapy was given along with wide-spectrum antimicrobial drug therapy (levofloxacin, trimethoprim-sulfamethoxazole, piperacillin-tazobactam, and fluconazole). After moderate improvement, the patient’s general condition worsened and prompted a new round of microbiologic tests, including those for mycobacteria. After acid-fast bacilli were detected in a bronchial aspirate and results of nucleic acid amplification (Amplicor; Roche, Basel, Switzerland) were positive for *M*. *tuberculosis* complex, she was given standard antituberculosis treatment. She died the next day.

Three blood cultures obtained 7 days before the death of the patient showed positive results in aerobic bottles but not in anaerobic bottles (BacT/ALERT SA; bioMérieux, Marcy l’Etoile, France). Subcultures spread onto blood agar yielded small white colonies of gram-positive bacilli, including branched forms, within 24 hours. One month earlier, the patient had been seen as an outpatient with intermittent fever, and unidentified gram-positive bacilli were observed in her blood culture.

Genetic sequencing of the first one third of the 16S rDNA gene (*1*) of the strain (GenBank accession no. EU370523) showed 99.8% identity with *M*. *abscessus*, *M*. *bolletii*, *M*. *chelonae*, and *M*. *massiliense*. To discriminate between these 4 species, a 723-bp fragment of the RNA polymerase β subunit (*rpoB*) gene (*2*) also was sequenced (GenBank accession no. EU370524). Sequencing showed 100% similarity with *M*. *massiliense*; the next most closely related species was *M*. *bolletii* (98.6% similarity).

An isolate of *M*. *tuberculosis* that was susceptible to all first-line antituberculosis drugs was recovered from this patient’s bronchial aspirate in both solid and liquid media. Immunocompromised patients, including those with organ transplants, are known to be prone to mycobacterial infections (*3*). Mixed mycobacterial infections also have been reported (*4*). This newly described species has been isolated from pulmonary fluids (*5*), blood (*6*), intramuscular injection sites (*7*), and surgical wounds (*8*). Because sequencing 16S rDNA does not differentiate *M*. *abscessus*, *M*. *bolletii*, *M*. *chelonae*, and *M*. *massiliense* (*5*), use of the *rpoB* sequence is crucial.

As reported for isolates of *M*. *massiliense* (*5*–*8*), our isolate was characterized by high MICs (*9*) (broth microdilution by using the Sensititer RGMYCO; Trek Diagnostic Systems Inc., Cleveland, OH, USA) to most of the antimicrobial drugs tested. The isolate was sensitive only to clarithromycin and amikacin and showed borderline sensitivity to linezolid (Table).

**Table:** Antimicrobial drug resistance pattern of the strain of  *Mycobacterium massiliense* isolated from the patient, Italy*

Drug	MIC (μg/mL)	Pattern
Amikacin	4	S
Amoxicillin/clavulanic acid	64/32	R
Cefoxitin	64	I
Ceftriaxone	>64	R
Ciprofloxacin	16	R
Clarithromycin	<0.12	S
Gatifloxacin	>8	R
Imipenem	32	R
Linezolid	8	S
Tobramycin	16	R
Trimethoprim/sulfamethoxazole	8/152	R

*S, sensitive; R, resistant; I, intermediate resistance.

Despite co-infection with *M*. *tuberculosis*, the patient’s death was likely caused by *M*. *massiliense* bacteremia. The patient died before the isolate was identified as a mycobacterium, and unfortunately, none of the drugs used empirically was active against this organism.

Only speculations can be made about how this patient acquired the *M*. *massiliense* infection. However, 5 months before her hospital admission, the patient had received a coxofemoral arthroprosthesis as a result of a fall and had since complained of generalized bone pain and had remained bedridden. Although in this case no proof exists, infection caused by rapidly growing mycobacteria after surgical intervention (*10*) is well known and should be considered.

*M*. *massiliense* has been distinguished from *M*. *abscessus* by sequencing of the *rpoB* gene (*2*). Because this technology is available in relatively few clinical laboratories, cases of infection with *M*. *massiliense* may be mistakenly attributed to *M*. *abscessus*. Although infections with *M. massiliense* may be underrecognized, reports of these infections are raising concern. The capacity of this bacteria to infect different body sites is further evidence for the pathogenic potential of a rapidly growing mycobacteria in human infections (*10*).
